# Targeting Oncometabolites in Peritoneal Cancers: Preclinical Insights and Therapeutic Strategies

**DOI:** 10.3390/metabo13050618

**Published:** 2023-04-30

**Authors:** Revathy Nadhan, Srishti Kashyap, Ji Hee Ha, Muralidharan Jayaraman, Yong Sang Song, Ciro Isidoro, Danny N. Dhanasekaran

**Affiliations:** 1 Stephenson Cancer Center, The University of Oklahoma Health Sciences Center, Oklahoma City, OK 73104, USA; revathy-nadhan@ouhsc.edu (R.N.); srishti-kashyap@ouhsc.edu (S.K.); jihee-ha@ouhsc.edu (J.H.H.); muralidharan-jayaraman@ouhsc.edu (M.J.); 2 Department of Cell Biology, The University of Oklahoma Health Sciences Center, Oklahoma City, OK 73104, USA; 3 Department of Obstetrics and Gynecology, Cancer Research Institute, College of Medicine, Seoul National University, Seoul 151-921, Republic of Korea; 4 Laboratory of Molecular Pathology and NanoBioImaging, Department of Health Sciences, Università del Piemonte Orientale, 28100 Novara, Italy; ciro.isidoro@med.uniupo.it

**Keywords:** cancer metabolism, metabolite, metabolome, oncometabolite, metabolomics, tumor microenvironment, peritoneal cancers, aerobic glycolysis

## Abstract

Peritoneal cancers present significant clinical challenges with poor prognosis. Understanding the role of cancer cell metabolism and cancer-promoting metabolites in peritoneal cancers can provide new insights into the mechanisms that drive tumor progression and can identify novel therapeutic targets and biomarkers for early detection, prognosis, and treatment response. Cancer cells dynamically reprogram their metabolism to facilitate tumor growth and overcome metabolic stress, with cancer-promoting metabolites such as kynurenines, lactate, and sphingosine-1-phosphate promoting cell proliferation, angiogenesis, and immune evasion. Targeting cancer-promoting metabolites could also lead to the development of effective combinatorial and adjuvant therapies involving metabolic inhibitors for the treatment of peritoneal cancers. With the observed metabolomic heterogeneity in cancer patients, defining peritoneal cancer metabolome and cancer-promoting metabolites holds great promise for improving outcomes for patients with peritoneal tumors and advancing the field of precision cancer medicine. This review provides an overview of the metabolic signatures of peritoneal cancer cells, explores the role of cancer-promoting metabolites as potential therapeutic targets, and discusses the implications for advancing precision cancer medicine in peritoneal cancers.

## 1. Introduction

Peritoneal malignancies pose significant clinical challenges due to their poor prognosis [1,2,3,4]. The peritoneum is a thin layer of squamous mesothelial cells that envelops the organs within the abdominal cavity. Primary peritoneal cancer (PPC) is a rare cancer that arises from the peritoneal lining itself. Major subtypes of PPCs include peritoneal mesothelioma, serous carcinomas, desmoplastic small round cell tumors, solitary fibrous tumors, pseudomyxoma peritonei, and primary peritoneal carcinoma of unknown origin. On the other hand, secondary peritoneal cancers (SPCs) or peritoneal carcinomatosis (PC) are more common and arise due to the spread of primary tumors such as colorectal, gastric, pancreatic, ovarian, breast, and lung cancers [1,4]. While primary and secondary peritoneal cancers are different in their origins, studies on SPCs can provide valuable insights into the biology of PPCs. This is due to the observation that SPCs shares many similarities with PPCs in terms of its histopathology and molecular features.

Metabolic reprogramming is a hallmark of malignant cancer growth, and peritoneal cancers are no exception [5,6,7]. With the closed anatomical barrier, the tumor microenvironment (TME) of peritoneal cancers has a significant impact on tumor growth, metastasis, and therapy resistance [8,9]. Understanding the role of cancer cell metabolism and cancer-promoting metabolites in the peritoneal tumor microenvironment (PTME) can provide new insights into the mechanisms that drive tumor progression and metastasis. PTME is a complex milieu where diverse cellular and molecular components coordinate to promote tumor progression and metastasis. The major components that define the PTME are cancer cells, cancer-associated fibroblasts (CAFs), immune cells, the extracellular matrix (ECM), and blood and lymphatic vessels. The crosstalk between these components determines the metabolic status of cancer cells and their response to therapeutic interventions [10,11]. 

Oncometabolites are those metabolites that are produced and accumulated in cancer cells due to altered metabolism and which can promote the growth and progression of tumors [12,13,14,15]. In this review, we employ the term ‘oncometabolites’ to refer to specific metabolites that play a direct and well-established role in cancer-related pathways. The classification of metabolites as oncometabolites is based on rigorous criteria, considering their functional significance and impact on tumorigenesis. Only metabolites that have been extensively studied and demonstrated to contribute to key aspects of cancer biology, such as cell proliferation, energy metabolism, and signaling pathways, are included in this classification. It is important to note that not all metabolites with altered concentrations in cancer cells are automatically classified as oncometabolites. The criteria for classifying metabolites as oncometabolites are based on a comprehensive analysis of existing literature, experimental evidence, and consensus within the scientific community. However, as the field of oncometabolites is evolving, some classifications may be subject to ongoing investigation and refinement. Cancer-promoting oncometabolites are key players in the metabolic reprogramming of cancer cells, and their identification and targeting can provide novel paradigms for tumor progression in peritoneal cancers [13,14,16]. Several such oncometabolites have been implicated in the pathogenesis and therapy resistance of both PPCs and SPCs [13]. Oncometabolites can also contribute to the development of tumor heterogeneity and can influence the response of cancer cells to therapy [17,18]. Oncometabolites such as fumarate, glucose, 2-hydroxyglutarate, lactate, succinate, sarcosine, glutamine, asparagine, and choline have also been identified as potential biomarkers for different cancers [16]. Targeting cancer-promoting metabolites holds great promise for the development of novel diagnostic, prognostic, and therapeutic strategies for peritoneal cancers [19]. Combination therapy with metabolic inhibitors and chemotherapy or immunotherapy may also be effective in overcoming chemoresistance and improving patient outcomes [20,21].

This review focuses on the potential of oncometabolites as therapeutic targets for managing peritoneal cancers. We examine the metabolic signatures of cancer cells, their crosstalk, and the role of oncometabolites in driving tumor progression. We also discuss the challenges involved in implementing novel therapeutic strategies, including the identification of effective metabolic inhibitors and the optimization of drug delivery to peritoneal cancers. Additionally, we explore the opportunities for precision cancer medicine that arise from targeting oncometabolites in peritoneal cancers, such as using metabolomic profiling to identify biomarkers for early detection and prognosis and developing personalized metabolic inhibitors based on individual tumor metabolic signatures. This review focuses specifically on peritoneal cancers, including both PPCs and SPCs, which originate from other sites and progress to peritoneal cancer through intraperitoneal metastasis. It does not cover cancers that metastasize to other sites. Overall, the review aims to provide insights into the current state of research on oncogenic metabolites in peritoneal cancers and their potential for improving outcomes for patients with peritoneal cancers.

## 2. Dysregulated Metabolism, Oncometabolites, and Peritoneal Cancer Pathogenesis

**Figure 1:**
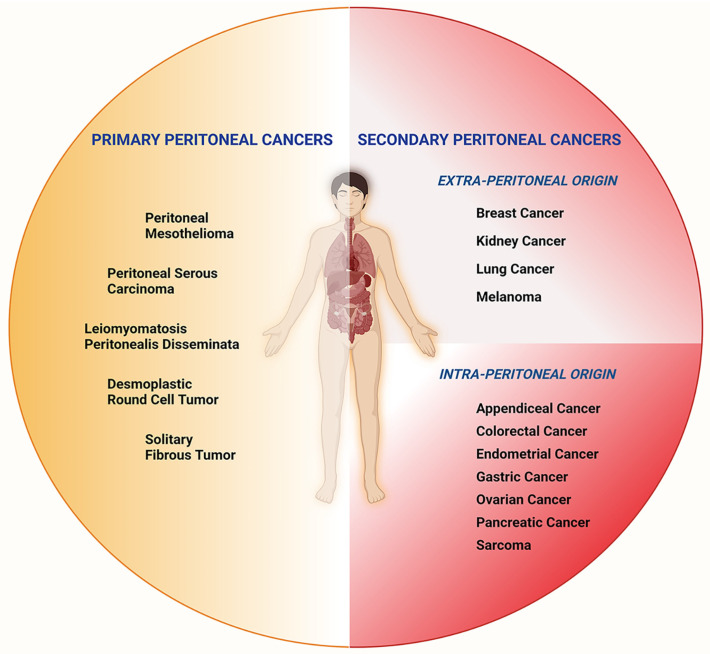
Subtypes of peritoneal cancers. Peritoneal Cancers can be categorized as primary and secondary, depending on their origins. Primary peritoneal cancers include peritoneal mesothelioma, peritoneal serous carcinoma, leiomyomatosis peritonealis disseminata, desmoplastic round cell tumors, and solitary fibrous tumors. Secondary peritoneal cancers of intra-peritoneal origin are appendiceal, colorectal, endometrial, gastric, ovarian, pancreatic cancers, and sarcomas. Peritoneal cancers of extra-peritoneal origin include breast, kidney, and lung cancers, as well as melanoma.

Cancer cells exhibit altered metabolism that contributes to their growth, survival, and resistance to therapy [22]. Metabolic alterations in cancer cells and their microenvironment result in the production and accumulation of oncometabolites that contribute to the development and progression of cancer [23]. Peritoneal cancers originate from the neoplastic transformation of the mesothelial cells that line the peritoneum or through the dissemination of malignant cells from other abdominal and extra-abdominal tumors that spread to the peritoneum (Figure 1). 

The incidence rate of primary peritoneal cancer has been determined to be 6.78 per million people, with higher rates among white individuals and lower rates among black individuals [24]. Comprehensive data on the percentage of occurrence for each of the subtypes of PPCs and SPCs are lacking due to the rarity of certain subtypes, variability in reporting, and the absence of centralized databases for comprehensive data dissemination. 

Nevertheless, the percentage of occurrences has been determined for a few subtypes of PPCs and SPCs [24]. The most common type of PPC is serous carcinoma of the peritoneum, accounting for approximately 10% of pelvic cancers. Malignant mesothelioma, although less common, is highly lethal, with pleural mesothelioma being the most prevalent type and peritoneal mesothelioma following it. Peritoneal metastasis, which involves the spread of cancer to the peritoneal cavity, is most frequently observed in ovarian cancers, occurring in 75% of cases at the time of diagnosis. It can also occur synchronously with the primary tumor in non-gynecological cancers or manifest during follow-up. Colorectal tumors demonstrate peritoneal spread in 5% to 10% of cases at diagnosis and metachronous spread in 20% to 50% of cases. Gastric cancers exhibit peritoneal dissemination in 14% of cases at initial presentation. Additionally, metastasis in the peritoneum can arise from cancers outside the abdomen, such as breast, lung, and melanoma, in 9% of cases. However, more detailed epidemiological studies are required to quantify the percentage of occurrences for other subtypes of PPCs and SPCs.

Although SPCs are distinct from the PPCs, they share the same or similar molecular features and metabolic alterations in the TME. Despite their heterogeneity, both PPCs and SPCs exhibit common molecular alterations in several key pathways. By identifying and targeting these pathways, it may be possible to develop effective therapies for peritoneal cancers. Additionally, the peritoneal environment itself plays a significant role in the development of peritoneal cancers, making it an important target for therapeutic intervention. Therefore, studying these cancers can provide a deeper understanding of the molecular mechanisms and metabolic pathways involved in peritoneal cancer pathophysiology. These insights can aid in the development of novel therapeutic approaches for these cancers. This section will discuss the metabolic pathways and oncometabolites of peritoneal cancers, primarily identified through the analyses of SPCs, and their potential as therapeutic targets in peritoneal cancers.

### 2.1. Glucose Metabolism 

One of the hallmarks of cancer is the ability to reprogram cellular metabolism to support tumor growth and survival [22,25]. Tumor cells heavily rely on the mitochondrial tricarboxylic acid (TCA) cycle, glycolysis, and pentose phosphate pathway (PPP) to meet the high demands for energy and biosynthesis during rapid cancer growth [26]. The TCA cycle, also known as the Krebs cycle, is a central metabolic pathway that plays a critical role in energy metabolism and biosynthesis. TCA cycle enzymes are frequently deregulated in cancer cells to meet the increased energy and biosynthetic needs of the rapidly proliferating cancer cells [27,28]. The oncometabolites derived from the TCA cycle, such as succinate, fumarate, and 2-hydroxyglutarate (2-HG), have emerged as key regulators of tumor growth and progression [29]. The TCA cycle enzymes play a critical role in the generation of these oncometabolites, with isocitrate dehydrogenase (IDH), fumarate hydratase (FH), and succinate dehydrogenase (SDH) being notable examples. IDH converts isocitrate to α-ketoglutarate (α-KG) in the TCA cycle. Mutations in IDH genes leading to the accumulation of 2-HG have been identified in a variety of cancers [30]. Similarly, mutations in FH and SDH genes, leading to the accumulation of oncometabolites fumarate and succinate, respectively, have been identified in certain cancers [31,32,33]. Elevated levels of succinate, fumarate, and 2-HG have been observed in many cancers that progress into PCs [32,34,35]. These oncometabolites have been shown to play a role in cancer growth and progression through various mechanisms including the inhibition of histone and DNA demethylases, the activation of hypoxia-inducible factor (HIF) signaling, and the modulation of epigenetic regulation [32,34]. 

In addition to altered metabolism involving the TCA cycle, most cancer cells manifest a shift in glucose metabolism towards aerobic glycolysis, which is characterized by the upregulation of glycolysis and the production of lactate [36,37,38]. This is a phenomenon where cancer cells preferentially use glycolysis for energy production, even in the presence of oxygen. This results in the production of lactate, which can create an acidic microenvironment that promotes cancer cell survival and proliferation. This metabolic shift, known as the Warburg effect, allows cancer cells to survive and proliferate in the hypoxic and nutrient-poor microenvironment of tumors [36,38,39]. The Warburg effect has been observed in both PPCs and SPCs, suggesting that it may be a common metabolic feature of peritoneal cancers. The deregulated glucose metabolism in peritoneal cancers has been well-documented in several studies. In peritoneal mesothelioma, for example, studies have shown the increased expression of glucose transporter 1 (GLUT1) and its utility as a prognostic factor [40,41,42]. This upregulation of glucose transporter and hexokinase enables the cancer cells to take up more glucose and convert it into glucose-6-phosphate, the first step in glycolysis. Enhanced glucose uptake by GLUT1 also augments the TCA cycle-mediated energy metabolism of the cancer cells. 

The overexpression of glycolytic enzymes such as lactate dehydrogenase (LDH) and pyruvate kinase (PK) has also been observed in both PPCs and SPCs [43,44,45,46,47]. LDH converts pyruvate to lactate, while PK converts phosphoenolpyruvate (PEP) to pyruvate. Both LDH and PK are key enzymes in the glycolytic pathway, and their upregulation leads to increased lactate production and reduced oxidative phosphorylation in cancer cells. Several metabolites from altered glucose metabolism have been identified as oncometabolites in different cancers [13]. Lactate is one of the metabolites from glucose metabolism that has been identified as an oncometabolite in many cancers. Elevated lactate levels in the serum have been associated with poor prognosis and postoperative complications in patients with peritoneal cancers [48]. Lactate not only provides energy for the cancer cells but also regulates the TME by promoting angiogenesis, immunosuppression, and ECM remodeling [38]. Several studies have established this glycolytic shift in the SPCs, leading to the accumulation of malignant ascites in the peritoneum. Metabolomic profiles of ascites from ovarian cancer patients have indicated elevated levels of glucose, suggesting an increased uptake of glucose by the cellular components of the PTME. Furthermore, the analysis of peritoneal lavage from gastric cancer patients with peritoneal metastasis has shown an elevation in the levels of glyceraldehyde-3-phosphate, indicative of increased aerobic glycolysis in gastric cancer patients [49]. Proteomic analyses of ascites from SPC patients have validated the glycolytic shift in these cancers, as evidenced by the increased expression of GLUT1 and the glycolytic enzyme, LDH, in peritoneal metastases of ovarian cancer [50]. 

Peritoneal cancer cells exhibit changes in the pentose phosphate pathway (PPP) in addition to the Warburg effect. The PPP produces nicotinamide adenine dinucleotide phosphate (NADPH), a crucial cofactor in antioxidant and nucleotide production that is essential for maintaining redox balance and protecting against oxidative stress. Studies have shown that the PPP is upregulated in both PPCs and SPCs, indicating that peritoneal cancers may be particularly susceptible to oxidative stress. PPP-derived NADPH is utilized to promote tumor growth by overcoming oxidative stress. The inhibition of glucose 6-phosphate dehydrogenase (G6PD) has been shown to reduce omental metastasis of ovarian cancer cells, thus suggesting a role of PPP in peritoneal metastasis of ovarian cancers [51,52]. A similar role of PPP pathway intermediates has been observed in peritoneal metastasis involving gastric, pancreatic, and colorectal cancers [49,53,54]. It has also been observed that the key enzymes of PPP such as G6PD, transaldolase, and transketolase are upregulated in pleural mesothelioma cell lines, which has a certain degree of similarity with the peritoneal mesothelioma [55]. Increased levels of glyceraldehyde-3-phosphate in the peritoneal lavage from gastric cancer patients with peritoneal metastasis are also suggestive of enhanced aerobic glycolysis and PPP in these gastric cancers [49].

The deregulated glucose metabolism in peritoneal cancers has important implications for diagnosis, treatment, and prognosis. Positron emission tomography (PET) and magnetic resonance imaging (MRI) are among the imaging techniques that exploit the increased glucose uptake of cancer cells as diagnostic markers [2,56,57]. The glucose analogue [^18^F]-2-deoxy-2-fluoro-D-glucose ([^18^F]-FDG) is a widely used radiotracer in oncological practice since its uptake is associated with various malignant processes. The unique metabolic phenotype of peritoneal cancer cells also presents an opportunity for targeted therapies. Several drugs that target glycolysis and the Warburg effect are currently being investigated for the treatment of cancer. For example, 2-deoxy-D-glucose (2-DG) is a glucose analog that inhibits glycolysis and has been shown to have antitumor effects in preclinical studies. Other drugs that target key enzymes in the glycolytic pathway, such as LDH inhibitors and PK inhibitors, are also under development. The deregulated glucose metabolism in peritoneal cancers may also have prognostic implications. Studies have shown that increased expressions of GLUTs and HKs are associated with poor prognosis in peritoneal mesothelioma. Elevated levels of LDH and glucose levels in the peritoneal fluid have also been found to be associated with poor prognosis in several types of cancers, including peritoneal mesothelioma, as well as SPCs including those of ovarian and gastric cancers. 

**Figure 2:**
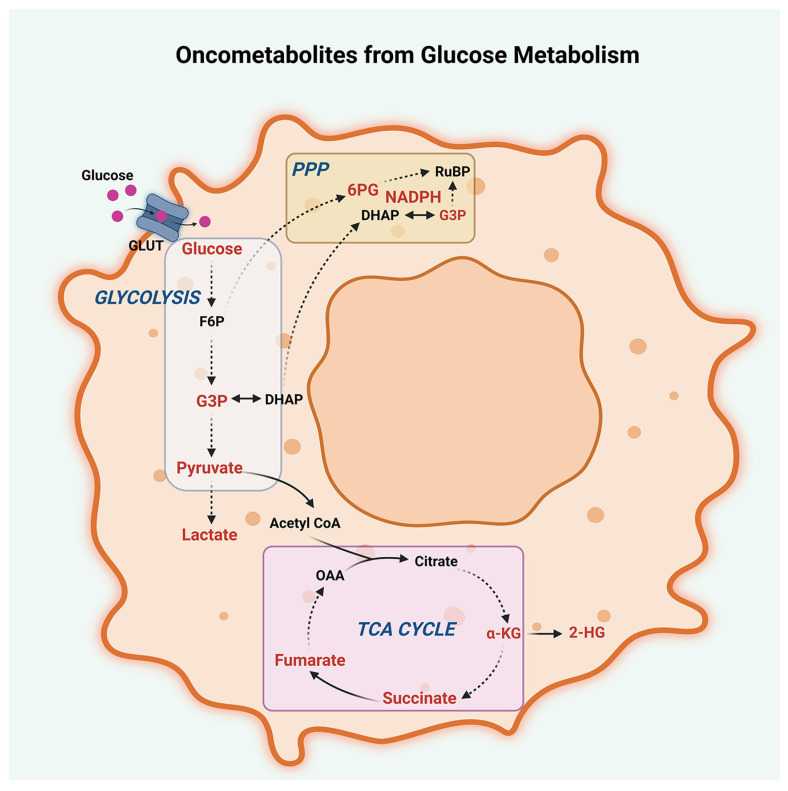
Oncometabolites from glucose metabolism. Oncometabolites derived from dysregulated glucose metabolism in peritoneal cancers, including glycolysis, the tricarboxylic acid cycle (TCA cycle), and the pentose phosphate pathway (PPP), are presented. Oncometabolites arising from deregulated TCA cycle include pyruvate, α-ketoglutarate (α-KG), succinate, fumarate, and 2-hydroxyglutarate (2-HG). Oncometabolites from dysregulated glycolytic shift include increased levels of glucose, glyceraldehyde-3-phosphate (G3P), pyruvate, and lactate. Oncometabolites derived from the PPP are nicotinamide adenine dinucleotide phosphate (NADPH), G3P, and 6-phosphogluconate (6PG). Other abbreviations are GLUT, Glucose Transporters; F6P, Fructose-6-phosphate; DHAP, Dihydroxyacetone phosphate; OAA, Oxaloacetate; and RuBP, Ribulose-1,5-bisphosphate.

In addition to their diagnostic and prognostic values, metabolic markers can also be used to monitor treatment response in peritoneal cancers. Changes in glucose metabolism and lactate levels can be used to assess the effectiveness of chemotherapy and targeted therapies. For example, decreases in glucose uptake and lactate levels after treatment with glycolytic inhibitors have been observed in preclinical studies. In conclusion, deregulated glucose metabolism is a hallmark of peritoneal cancers and plays an important role in tumor growth and survival (Figure 2). 

### 2.2. Lipid Metabolism

**Figure 3:**
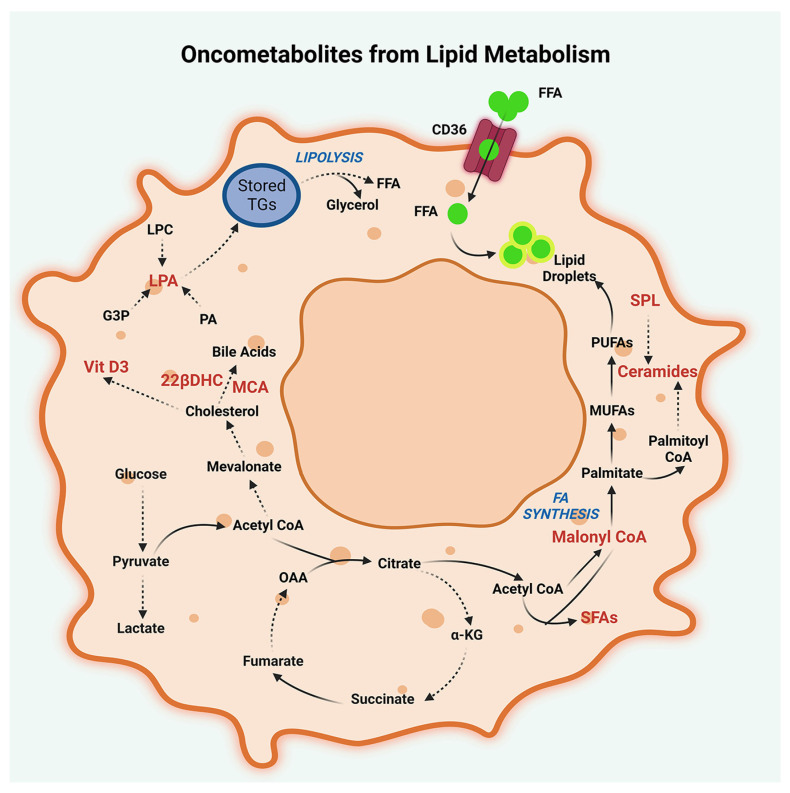
Oncometabolites from lipid metabolism. Lipid oncometabolites, generated by dysregulated metabolism of cholesterol, fatty acids, and triglycerides in peritoneal cancers are illustrated. Oncometabolites resulting from deregulated cholesterol metabolism are muricholic acid (MCA), 23-lactone, 22β-dihydroxy cholesterol (22βDHC), and 1,25-dihydroxyvitamin D3-26, 23-lactone (Vitamin D3). Oncometabolites generated by deregulated fatty acid metabolism include malonyl CoA, tridecanoic acid, tetradecanoic acid, and octadecanoic acid. Deregulated fatty acid metabolism also results in the production of signaling lipids such as ceramide (18:1), lysophosphatidic acid (LPA), and sphingolipids (SPL). Additionally, potential oncometabolites resulting from lipid metabolism in peritoneal cancers include the metabolites derived from the lipolysis of storage triglycerides (TG). The metabolites denoted by abbreviations are FFA, Free Fatty Acids; CD-36, Fatty acid translocase CD36; PUFAs, Polyunsaturated fatty acids; MUFAs, Monounsaturated fatty acids; α-KG, α-ketoglutarate; OAA, Oxaloacetate; PA, Phosphatidic acids; SFA, Saturated fatty acids; and G3P, Glyceraldehyde-3-phosphate.

Lipid metabolism is another important aspect of cancer metabolism that can be targeted for therapy (Figure 3). Lipids, such as triglycerides, sterols, sphingolipids, and phospholipids, are used as an energy source by cancer cells, as well as the constituents of biological membranes. Metabolomic analysis of peritoneal lavage and malignant ascites from metastasis of SPCs have shown that lipid metabolism is deregulated in PCs [49,58]. Specifically, liquid chromatography-mass spectrometry (LC-MS) analysis of peritoneal lavage fluid from gastric cancer patients has revealed an upregulation of lipid profile, which includes triglycerides, glycerol monoesters, and fatty acids such as tridecanoic acid, octadecanoic acid, and tetradecanoic acid, pointing to an enhanced lipid turnover in PCs [49].

In vitro studies have shown that intercellular metabolic crosstalk is involved in the reprogramming of lipid metabolism. This is evidenced by the release of fatty acids by both CAFs and adipocytes into the PTME, facilitating the fatty acid uptake by the cancer cells [59,60]. Co-culture studies with ovarian cancer cells and adipocytes have indicated that the cancer cells stimulate the release of fatty acids from adipocytes, which are readily taken up by the cancer cells [60]. Using this model system that emulates PTME, it has been shown that cancer cells stimulate the β-adrenergic-receptor-mediated activation of the protein kinase-A signaling pathway in adipocytes to promote lipolysis of stored triglycerides into glycerol and free fatty acids. Released fatty acids are taken up by the cancer cells for accelerated tumor growth [60].

Cholesterol metabolism is also dysregulated in PCs. Malignant ascites obtained from chemoresistant ovarian cancer patients were assessed for their metabolomic profiles and have reported elevated levels of cholesterol metabolic intermediates such as muricholic acid, 23-lactone, 22β-dihydroxy cholesterol, and 1,25-dihydroxyvitamin D3-26, 23-lactone (Vitamin D3) that serve as oncometabolites. These data indicate that cancer cells metabolize cholesterol to promote the synthesis of bile acids and steroid hormones that, in turn, favor cancer cell proliferation and invasion [58]. An analysis of ascitic fluid was performed to compare the proteomic-metabolomic profiles of ovarian cancer and cirrhosis patients. This study revealed the presence of metabolic derivatives of signaling lipids, such as ceramide (18:1) and lysophosphatidic acid (LPA) in the ascites derived from ovarian cancer patients, but not in those from cirrhosis patients [50]. LPA is a well-characterized oncometabolite that has been reported to play a role in promoting the proliferation and metastasis of ovarian cancer. In addition, LPA present in the ascites has been shown to stimulate a pseudo-hypoxic response in ovarian cancer cells and CAFs to enhance aerobic glycolysis [61].

Enhanced lipid metabolism is known to play a crucial role in the survival and growth of cancer cells due to their close association with the endoplasmic reticulum (ER) and the Golgi apparatus. These organelles are essential for protein synthesis, processing, and transport within the cell. The ER is responsible for the synthesis and folding of membrane and secretory proteins, as well as the synthesis of lipids such as phospholipids and cholesterol. Interestingly, the first step in cholesterol biosynthesis occurs within the ER. Meanwhile, the Golgi is critical for modifying, sorting, and transporting lipids, as well as synthesizing complex glycolipids and glycoproteins that are involved in various cellular functions such as cell signaling. Consequently, the increased function of ER and Golgi in cancer cells is closely associated with enhanced lipid metabolism. However, the specific roles of ER and Golgi in PPCs and SPCs are yet to be fully elucidated.

### 2.3. Amino Acid Metabolism

Amino acid metabolism is crucial for cancer cell survival and proliferation, and major amino acid metabolisms that correlate with cancer progression include glutamine, serine-glycine, branched-chain amino acids, arginine, tryptophan, histidine, and methionine. These amino acids serve as sources for the synthesis of non-essential amino acids, as well as precursors for glucose, lipid, and nucleic acid synthesis, and also act as signaling molecules that activate the mTOR pathway. Additionally, amino acid metabolic intermediates such as S-adenosylmethionine (SAM), derived from the methionine cycle, contribute to epigenetic regulation and enter the TCA cycle as keto acids. Glutathione, a metabolite of glutamine, plays a cardinal role in the redox balance in cancer cells, and glutamine metabolism serves as the prime core that links to glucose metabolism, lipid precursors, amino acid metabolism, and nucleotide synthesis.

Dysregulated amino acid metabolism has been identified as a critical mechanism underlying peritoneal cancer pathobiology. LC-MS analysis of peritoneal lavage fluid from gastric cancer patients with peritoneal metastases has identified several upregulated amino acid metabolites including 3-methyl alanine, α-amino butyric acid, and glutamyl alanine. These oncometabolites have been shown to have the potential as diagnostic biomarkers for peritoneal cancers [49]. Of these oncometabolites, α-amino butyric acid has been correlated with oxidative stress-induced glutathione metabolism and has been observed at increased levels in several cancers that can metastasize to the peritoneum, such as ovarian and colorectal cancers. It has also been identified as a marker of worse prognosis in colorectal cancers [62,63]. The altered metabolism of methionine has been associated with upregulated levels of homocysteine, which has been identified as a prospective diagnostic biomarker for several cancers, including lung, breast, and gastric cancers [64,65,66]. 

Glutamine is the most plentiful amino acid in the body and plays a cardinal role in cancer cell bioenergetics and biosynthetic pathways. A shift towards glutamine metabolism has been shown to play a critical role in tumor growth and invasive metastasis of ovarian cancers [67]. Glutamine is involved in redox homeostasis and the detoxification of xenobiotics through its pivotal role in glutathione metabolism. Glutamine metabolism is driven by the expression of genes such as glutaminases, glutamate dehydrogenases, and glutamate oxaloacetate transaminases, whose expression is enhanced in cancer cells that rely on glutamine metabolism for redox homeostasis, TCA cycle, and cellular biosynthetic pathways [68,69]. Glutamine metabolism generates several oncometabolites that promote tumor growth and immune suppression. For instance, α-KG, a metabolite of glutamine metabolism, is a critical cofactor for the activity of HIF that promotes angiogenesis and tumor growth [70]. 

The dysregulation of amino acid metabolism in peritoneal cancers is due not only to changes in cancer cells but also to the contributions of the TME. Intercellular metabolic crosstalk underlying glutamine metabolism has been observed in the TME of multiple cancers. CAFs and adipocytes in the PTME can promote glutamine metabolism in cancer cells by synthesizing and releasing glutamine into the TME [71,72]. Specifically, CAFs stimulate glutamine metabolism in cancer cells by coupling the TCA cycle and glutamine synthesis and its subsequent release into the TME through multiple mechanisms. Glucose and lactate levels in the TME drive glutamine synthesis in ovarian CAFs through TCA-cycle-generated acetyl-CoA and TCA metabolites such as oxaloacetic acid. Glutamine is then released into the TME for uptake by the cancer cells through specific transporters [71]. In peritoneal metastases of colorectal cancer, cancer cells avidly uptake glutamine from the PTME, leading to a glutamine shortage. Adipocytes in the TME respond by enhancing the endogenous glutamine synthesis, which is then released into the PTME to replenish the glutamine pool. This glutamine is taken up by the cancer cells through specific transporters and is converted into glutamate by glutaminases through glutaminolysis. Thus, glutamine is utilized by cancer cells to facilitate bioenergetic and biosynthetic pathways [72]. These observations highlight how the TME can dynamically modulate amino acid metabolism in peritoneal cancers.

Tryptophan metabolism is yet another critical amino acid metabolic pathway that is dysregulated in many cancers, including peritoneal cancers [73]. Tryptophan is metabolized through the kynurenine pathway, which generates several oncometabolites that promote tumor growth and immune suppression. Tryptophan metabolites generated by indoleamine-2,3-dioxygenase (IDO) and tryptophan-2,3-dioxygenase (TDO) have been shown to promote tumor growth and immune evasion in peritoneal cancers [74,75]. IDO and TDO are enzymes that catalyze the first step of the kynurenine pathway, leading to the generation of kynurenine and other immunosuppressive metabolites [76,77]. IDO has been shown to promote tumor growth and immune evasion in many SPCs by inhibiting T cell activation and promoting the differentiation of regulatory T cells [78,79]. TDO has been implicated in the immune evasion of colorectal cancer by upregulating immune checkpoint molecules such as programmed death ligand-1 (PD-L1), which inhibits T cell function and promotes tumor growth [80]. Kynurenine, derived from the activities of IDO and TDO, promotes tumor growth and metastasis by suppressing immune surveillance and facilitating the recruitment of immunosuppressive cells to the TME [81]. Kynurenine is an immunomodulatory molecule that can suppress T cell activity and promote the differentiation of regulatory T cells and myeloid-derived suppressor cells (MDSCs) [78]. Furthermore, the microbiota in the gut lumen can modulate tryptophan metabolism and kynurenine pathway activation, leading to the production of oncometabolites that promote tumorigenesis and immune suppression. For instance, gut bacteria have been shown to promote kynurenine pathway activation and the production of immunosuppressive metabolites in colorectal cancer [80].

**Figure 4:**
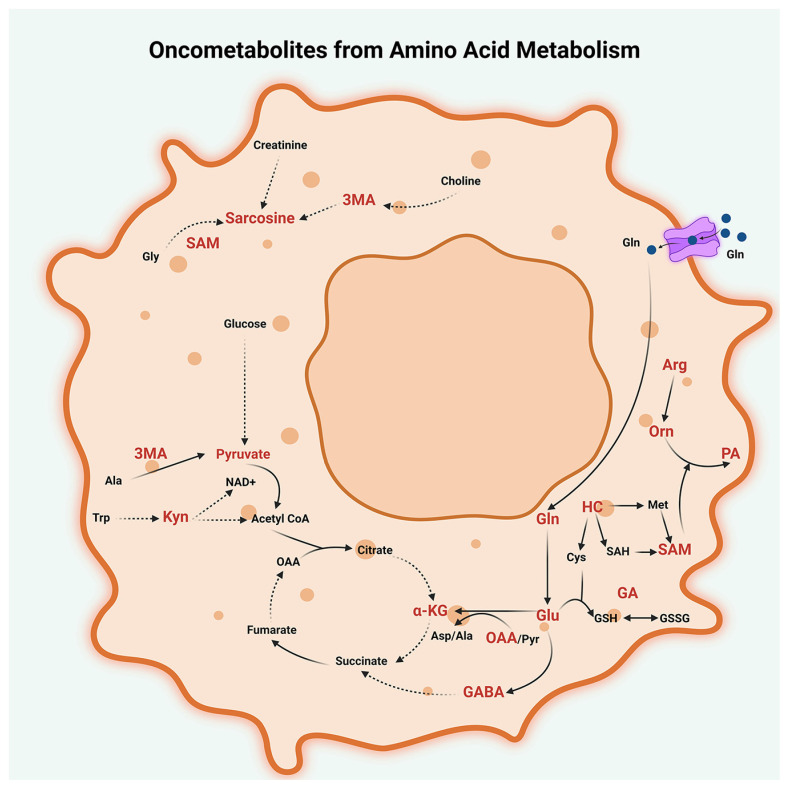
Oncometabolites from amino acid metabolism. Dysregulated metabolism of glutamine (Gln), glutamic acid (Glu), tryptophan (Trp), methionine (Met), alanine (Ala), arginine (Arg), and glycine (Gly) generate several oncogenic metabolites in peritoneal cancers. These metabolites include glutamine (Gln), glutamate (Glu), oxaloacetate (OAA), glutamyl alanine (GA), and α-ketoglutarate (α-KG) from glutamine (Gln) metabolism; α-amino butyric acid (GABA) from glutamine-glutamate metabolism; 3-methyl alanine (3MA) from alanine (Ala) metabolism; glutamyl alanine (GA) from glutamine-alanine metabolism; sarcosine from glycine (Gly) metabolism; homocysteine (HC); and S-adenosyl methionine (SAM) from methionine metabolism; ornithine (Orn), polyamines (PA), and depletion of arginine (Arg) from arginine metabolism; and kynurenine (Kyn) from tryptophan (Trp) metabolism; and glutathione (GSH) from Cysteine (Cys)-glutamate-glycine metabolism. Other abbreviations used are SAH, S-adenosyl homocysteine; Cys-Cysteine; GSH, glutathione-reduced; GSSG, Glutathione-oxidized; Pyr, Pyruvate; OAA, Oxaloacetate; Asp, Aspartate; and NAD+, Nicotinamide adenine dinucleotide.

The metabolism of arginine and histidine is also dysregulated in some peritoneal cancers. Arginine metabolism is critical for the function of immune cells, and the depletion of arginine by arginase-expressing MDSCs can inhibit anti-tumor immune responses [82]. In summary, the dysregulation of amino acid metabolism plays a critical role in peritoneal cancer pathobiology, and different amino acids can contribute to tumor growth and TME regulation through a variety of mechanisms. Targeting amino acid metabolism may represent a promising strategy for the development of new diagnostic biomarkers and therapeutic approaches for peritoneal cancers (Figure 4).

### 2.4. Nucleotide Metabolism

Nucleotides, which include purines (adenine and guanine) and pyrimidines (thymine, uracil, and cytosine), are the building blocks of cellular genetic material and are essential for highly proliferating cancer cells. Nucleotide metabolism, involving purine and pyrimidine pathways, is required for DNA and RNA synthesis, cellular signaling, and regulating cellular metabolism and enzyme activity. Deregulated nucleotide metabolism, which is characterized by alterations in the synthesis and utilization of nucleotides, is another hallmark of cancer metabolism that plays a crucial role in peritoneal cancers. The de novo synthesis of nucleotides is essential for DNA replication and repair, as well as for the production of RNA and other nucleic acid derivatives [83]. In cancer cells, nucleotide metabolism is dysregulated, leading to the overproduction or accumulation of oncometabolites that contribute to the development and progression of cancer. The de novo pathways of purine and pyrimidine synthesis play a crucial role in cancer cell proliferation, as they provide the necessary precursor molecules for nucleotide synthesis. The purine de novo pathway begins with the synthesis of phosphoribosyl pyrophosphate (PRPP), a molecule derived from glucose metabolism, and the enzyme PRPP synthetase. The pathway then goes through several steps, including the synthesis of inosine monophosphate (IMP) from PRPP, and the conversion of IMP to adenosine monophosphate (AMP) and guanosine monophosphate (GMP) via the actions of the enzymes adenylosuccinate synthestase (ADSS) and inosine 5′-monophosphate dehydrogenase (IMPDH), respectively. Peritoneal cancers, including ovarian cancer, have been found to exhibit alterations in purine metabolism, which contribute to their pathogenesis and progression. Several studies have shown that the enzymes involved in the purine de novo synthesis pathway, including PRPP synthetase, ADSS, and IMPDH, are upregulated in ovarian cancer and other peritoneal cancers [84,85]. Additionally, there is evidence of altered purine salvage pathways, with increased expression of the enzyme hypoxanthine phosphoribosyltransferase (HPRT) in ovarian cancer cells. These alterations in purine metabolism have been shown to contribute to cancer cell proliferation, survival, and drug resistance, making them an attractive target for cancer therapy. Inhibitors of enzymes involved in purine metabolism, such as IMPDH inhibitors and HPRT inhibitors, have shown promising results in preclinical studies and are being investigated for their potential therapeutic use in peritoneal cancers, including ovarian cancer [84].

Alterations in pyrimidine metabolism have been shown to contribute to cancer cell proliferation, survival, and drug resistance. The de novo pyrimidine synthesis pathway is essential for the production of pyrimidine nucleotides, which are required for DNA and RNA synthesis. One oncometabolite that has been implicated in peritoneal cancers is dihydroorotate (DHO), an intermediate in the de novo synthesis of pyrimidine nucleotides [83]. DHO is converted to orotate by dihydroorotate dehydrogenase (DHODH), an enzyme that is upregulated in many types of cancers, including peritoneal cancers [86]. The accumulation of DHO and the overexpression of DHODH contribute to the development of peritoneal cancers by promoting DNA synthesis and cell proliferation [83,86]. Enzymes involved in the salvage pathway, such as uridine-cytidine kinase (UCK) and thymidine kinase 1 (TK1), have also been found to be upregulated in ovarian cancer. Therefore, targeting enzymes involved in pyrimidine metabolism, such as DHODH and thymidylate synthetase (TYMS), has emerged as a promising approach for the development of novel cancer therapies for peritoneal cancers, including ovarian cancer [87].

In addition, the oncometabolite 5-phosphoribosyl-1-pyrophosphate (PRPP) has been shown to promote tumor growth and survival in peritoneal cancers [88]. PRPP is an intermediate in the synthesis of purine and pyrimidine nucleotides and is essential for the maintenance of nucleotide pools in cancer cells. In peritoneal cancers, PRPP is upregulated and contributes to the overproduction of nucleotides, which are required for DNA synthesis and cell proliferation [88].

Furthermore, recent studies have identified the oncometabolite nicotinamide adenine dinucleotide (NAD+) as a critical regulator of nucleotide metabolism in cancer cells [89]. NAD+ is a cofactor that plays a crucial role in energy metabolism and DNA repair. In peritoneal cancers, NAD+ is dysregulated, leading to the overproduction of oncometabolites such as nicotinamide mononucleotide (NMN) and nicotinamide adenine dinucleotide phosphate (NADP), which promotes tumor growth and survival [89]. The overexpression of enzymes involved in nucleotide metabolism is another mechanism that contributes to the development and progression of peritoneal cancers. For example, TYMS, an enzyme involved in the synthesis of thymidine, is upregulated in peritoneal cancers and is associated with poor prognosis [90]. Similarly, studies have shown that the expression DHODH, which is involved in orotate synthesis, is upregulated in many cancers including peritoneal cancers [86].

**Figure 5:**
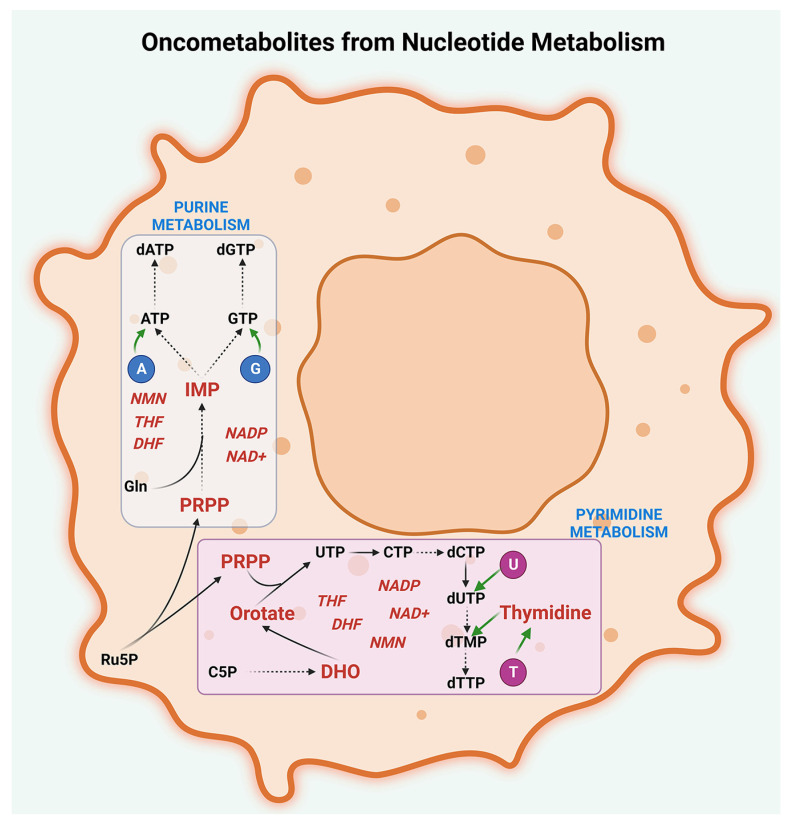
Oncometabolites from nucleotide metabolism. Oncogenic intermediates are generated by the deregulated metabolism of purines, pyrimidines, and their cofactors in peritoneal cancers. Oncometabolites resulting from purine metabolism include inosine monophosphate (IMP) and 5-phosphoribosyl-1-pyrophosphate (PRPP) while oncometabolites generated by pyrimidine metabolism include dihydroorotate (DHO), orotate, PRPP, and thymidine. The major cofactors involved in purine and pyrimidine metabolisms that act as oncometabolites are nicotinamide mononucleotide (NMN), nicotinamide adenine dinucleotide (NAD+), nicotinamide adenine dinucleotide phosphate (NADP), dihydrofolate (DHF), and tetrahydrofolate (THF). The abbreviations denote A, Adenine; G, Guanine; U, Uracil; T, Thymine; C5P, Carbamoyl-5-phosphate; UTP, Uridine triphosphate; CTP, Cytidine triphosphate; dCTP, Deoxycytidine triphosphate; dUTP-Deoxyuridine triphosphate; dTMP-Deoxythymidine monophosphate; dTTP, Deoxythymidine triphosphate; Ru5P, Ribulose-5-phosphate; Gln, Glutamine; ATP, Adenosine triphosphate; GTP, Guanosine triphosphate; dATP, Deoxyadenosine triphosphate; and dGTP, Deoxyguanosine triphosphate.

Overall, these studies suggest that the dysregulation of nucleotide metabolism is a critical process in the development and progression of peritoneal cancers (Figure 5). 

Targeting nucleotide metabolism, either by inhibiting the enzymes involved in nucleotide synthesis or by reducing the production of oncometabolites, is a promising approach for the development of novel cancer therapies. Further research is needed to identify additional targets and develop more precise and personalized strategies for targeting nucleotide metabolism in peritoneal cancers.

## 3. Oncometabolites and Precision Cancer Medicine

**Figure 6:**
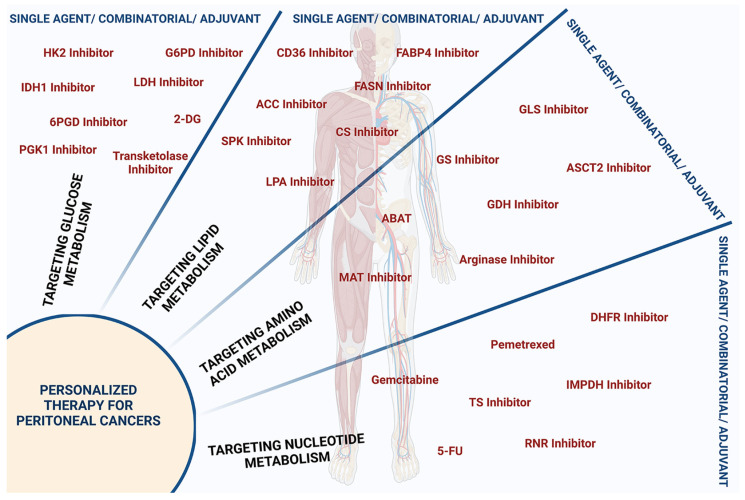
Protein targets influencing oncometabolites for personalized peritoneal cancer therapy. Metabolite inhibitors employed for personalized therapy of peritoneal cancers either as single agent or as component of combinatorial therapy or as an adjuvant are presented. Inhibitors targeting glucose metabolism include inhibitors of hexokinase-2 (HK-2 Inhibitor), glyceraldehyde-6-phosphate dehydrogenase (G6PD Inhibitor), isocitrate dehydrogenase-1 (IDH1 Inhibitor), lactate dehydrogenase (LDH Inhibitor), 6-phosphogluconate dehydrogenase (6PGD Inhibitor), phosphoglycerate kinase-1 (PGK1 Inhibitor), and transketolase, as well as 2-deoxy glucose (2-DG). Inhibitors targeting lipid metabolism include inhibitors of fatty acid translocase CD36 (CD36 Inhibitor), fatty acid binding protein 4 (FABP4 Inhibitor), fatty acid synthase (FASN Inhibitor), acetyl-CoA-carboxylase (ACC Inhibitor), ceramide synthase (CS Inhibitor), and sphingosine kinase I (SPK Inhibitor), and lysophosphatidic acid (LPA Inhibitor). Inhibitors targeting dysregulated amino acid metabolism include inhibitors of glutaminase (GLS Inhibitor), glutamine synthetase (GS Inhibitor), amino acid transporter ASCT-2 (ASCT-2 Inhibitor), glutamate dehydrogenase (GDH Inhibitor), arginase, methionine adenosyl transferase (MAT Inhibitor), and 4-aminobutyrate aminotransferase (ABAT). Inhibitors targeting nucleotide metabolism include inhibitors of dihydrofolate reductase (DHFR), inosine monophosphate dehydrogenase (IMPDH), ribonucleotide reductase (RNR), thymidylate synthase (TS), and folate Inhibitor, as well as nucleoside analogs such as 5-fluorouracil (5-FU) and 2′, 2′-difluoro 2′deoxycytidine or gemcitabine.

Precision cancer medicine aims to tailor treatment to individual patients based on the specific characteristics of their cancer, including their genetic, epigenetic, and metabolic profiles. Defining the metabolites that are generated as a result of altered metabolic pathways in peritoneal cancers will be crucial for developing precision cancer medicine strategies for these cancers. Targeting specific oncometabolites can potentially disrupt key signaling pathways that are essential for cancer cell survival and proliferation, leading to the development of effective targeted therapies [21,91]. Moreover, identifying specific oncometabolites in peritoneal cancers can provide valuable information to physicians for developing personalized treatments based on the specific molecular profile of each patient’s tumor that can target the altered metabolic pathways altered in their cancer. This approach can potentially improve treatment outcomes and reduce the risk of adverse effects associated with conventional chemotherapy. With the characterization of oncometabolites, these goals have been achieved to a certain extent in many peritoneal cancers (Figure 6).

Several studies investigated the potential of oncometabolites as therapeutic targets in peritoneal cancers by focusing on the metabolic pathways altered in cancer cells [92,93,94]. The unique metabolic phenotype of peritoneal cancer cells also presents an opportunity for targeted therapies. Studies interrogating SPCs have shown that targeting cancer metabolism by inhibiting oncometabolites can be a promising approach for effective combinatorial or adjuvant therapy for PCs. Studies have identified that increased glucose and glutamine metabolism, as well as elevated lipid synthesis, played significant roles in promoting the growth and survival of cancer cells in the PTME. Studies have also suggested that targeting these metabolic pathways, and the oncometabolites they produce, could improve the effectiveness of chemotherapy in peritoneal cancers. Numerous clinical and preclinical studies have confirmed the effectiveness of targeting certain oncometabolites and their related pathways. Table 1 provides a summary of some of the studies that have been validated.

### 3.1. Targeting Glucose Metabolism

Several studies have demonstrated the potential of targeting glucose metabolism in cancer cells. Metabolic intermediate inhibitors have been used to treat peritoneal cancers since 1964, when 2-DG, a glycolytic inhibitor, was tested on cancer cells derived from patient ascites [124]. Furthermore, tumor metabolism was analyzed to predict the outcome of surgical procedures such as upfront debulking surgery in ovarian cancers and PPCs or post-chemotherapy [56,125,126]. Given the pivotal role of glucose metabolism in the PTME, targeting the oncogenic metabolites from these pathways could be a promising strategy for cancer therapy [52]. Targeting cancer-promoting metabolites from the TCA cycle, glycolytic pathway, and PPP pathway represents a promising therapeutic strategy for the treatment of peritoneal cancers. Several compounds targeting the key enzymes of the TCA cycle, glycolysis, and the PPP pathway have been identified as potential therapeutic agents, and their efficacy has been demonstrated in preclinical models [28].

Although cancer cells reprogram their metabolism towards the glycolytic pathway, recent studies demonstrated the potential of targeting TCA cycle enzymes for many cancers [26,127]. Although their efficacy remains to be fully investigated in PPCs, their effectiveness as therapeutic targets has been well established in SPCs or cancers that progress to SPCs. For instance, the mitochondrial enzyme IDH has been shown to play a critical role in regulating cancer metabolism by producing the oncometabolite 2-HG. 2-HG has been shown to promote the growth, survival, and metastasis of colorectal, pancreatic, cholangiocarcinoma, ovarian, and gastric cancer cells [128,129,130,131]. Targeting IDH1, thereby inhibiting the production of 2-HG, has been shown to prevent peritoneal metastasis of ovarian cancers by inducing senescence [99,132]. More importantly, a small molecular inhibitor of IDH1 has been shown to provide a survival advantage to cholangiocarcinoma patients [133]. 

Several studies have investigated the use of drugs that target the glycolytic pathway in peritoneal cancers. Using a mouse model of PPCs, it has been observed that treatment with 2-DG results in a significant reduction in tumor growth and increased survival of animals, likely due to the inhibition of glycolysis and the resulting decrease in energy production within the cancer cells [134]. Another study has shown that 2-DG in combination with metformin could inhibit glucose uptake and reduce cell proliferation in the ovarian cancer cell lines, indicating that targeting glucose metabolism could be a promising approach for the treatment of peritoneal cancers [95]. 

One of the most widely studied targets in the glycolytic pathway is the enzyme HK2, which catalyzes the first step in glucose metabolism. The inhibition of HK2 has been shown to impair glycolysis and lead to decreased cell proliferation and tumor growth in various cancer types. The inhibition of HK2 using 3-bromopuryuvate has been shown to induce cytostatic and cytotoxic effects in both in vitro and in vivo models of peritoneal mesothelioma [96]. The efficacy of targeting HK2 has also been well characterized in cancers that can give rise to SPCs [135,136]. Likewise, small molecule inhibitors of the glycolytic enzyme LDH have been shown to reduce lactate production and inhibit tumor growth in preclinical models of different SPCs [97,137,138]. 

The PPP is another attractive target for cancer therapy, as it plays a critical role in the production of nucleotides and antioxidants that are necessary for cancer cell survival and proliferation. The rate-limiting enzyme in PPP is G6PD, which converts glucose-6-phosphate into 6-phosphogluconolactone, accompanied by NADPH production. Through its key role in PPP, G6PD has been shown to play a pleiotropic role in cancer genesis and progression [139]. Inhibition of the PPP enzyme, G6PD, has been shown to reduce tumor growth in preclinical models of breast, ovarian, and pancreatic cancers [52,100,140]. Another enzyme that plays a central role in PPP is 6-phosphogluconate dehydrogenase (6PGD), which converts 6-phosphogluconate (6-PG) into ribulose-5-phosphate (Ru5P) with the generation of NAPDH. Inhibitors of 6PGD and transketolase have been shown to have antitumor activity in preclinical models of ovarian and hepatocellular carcinoma [52,101,141]. 

Targeting cancer-promoting metabolites from the TCA cycle, glycolytic pathway, and PPP pathway represents a promising combinatorial therapeutic strategy for the treatment of peritoneal cancers. For instance, in peritoneal metastasis of gastric cancers, combinatorial therapy involving 5-fluorouracil (5-FU) and shRNA-mediated inhibition of phosphoglycerate kinase-1, which phosphorylates BECLIN-1 to induce autophagy, augments the cytotoxic effects of 5-FU [98]. While there is still much to be learned about the metabolic landscape of peritoneal tumors, preclinical studies have demonstrated the efficacy of several compounds targeting key enzymes in these pathways. Further investigation of these compounds in clinical trials may lead to the development of novel, effective therapies for peritoneal cancers. In addition to drug-based approaches, dietary interventions have also been explored as a way to target glucose metabolism in peritoneal cancers [142]. For example, a ketogenic diet has been associated with decreased tumor growth and increased patient survival, likely due to the reduction in glucose availability to the cancer cells and the resulting shift in the cells’ energy metabolism.

### 3.2. Targeting Lipid Metabolism

Targeting lipid metabolism has shown promise as a therapeutic strategy for peritoneal cancers. One approach is to inhibit fatty acid uptake by cancer cells. Several preclinical studies have demonstrated the efficacy of targeting fatty acid transporters such as fatty acid translocase, CD36, and fatty acid binding protein 4 (FABP4) in reducing tumor growth in various cancer types [102,143,144,145]. In addition, targeting lipid metabolism enzymes such as acetyl-CoA carboxylase (ACC) and fatty acid synthase (FASN) has also shown promise in reducing cancer growth [93,103,146,147]. This is more pronounced in the case of FASN, a key enzyme in fatty acid synthesis. Increased expression of FASN has been correlated with chemoresistance to drugs such as cisplatin in ovarian and lung carcinomas, doxorubicin or 5-FU in breast cancers, and gemcitabine in pancreatic cancers [148]. FASN inhibitors have been shown to synergistically sensitize cancer cells to numerous chemotherapy drugs [149] Under conditions of chemotherapeutic stress, FASN inhibits apoptosis by promoting the synthesis of fatty acids for energy production through β-oxidation, thereby offering pro-survival signals to the cancer cells. Inhibitors of FASN, such as orlistat and cerulenin, have shown anticancer activity in preclinical studies and are being evaluated in clinical trials [149]. 

Furthermore, targeting the sphingolipid metabolism pathway, which is involved in the synthesis of ceramides and other bioactive lipids, has also shown potential for cancer therapy [106]. Several sphingolipid metabolism inhibitors, such as ceramide synthase inhibitors and sphingosine kinase inhibitors, have been developed and are currently under investigation for cancer treatment [107,150]. Using preclinical animal models, it has also been shown that targeting LPA signaling is a potential therapeutic strategy in ovarian cancer [61,151]. Furthermore, lipid metabolic intermediates, such as sphingolipids and LPA, which serve as signaling lipids, confer chemoresistance toward numerous therapeutic agents by promoting diverse adaptive rescue survival signaling cascades [152]. Further research into targeting lipid metabolism may provide additional therapeutic options for peritoneal cancers.

Clinical studies have also identified components of lipid metabolism as potential biomarkers for predicting treatment response and prognosis in peritoneal cancers. For example, elevated levels of FABP1 and FASN have been shown to predict poor prognosis in patients with many different SPCs, such as those of gastric cancer [103,153]. 

### 3.3. Targeting Amino Acid Metabolism

Targeting amino acid metabolism has also emerged as a promising strategy for cancer therapy, including peritoneal cancers. Several amino acid metabolic pathways have been identified as potential targets, including the glutamine and methionine pathways. Glutamine metabolism is an attractive target for cancer therapy due to its crucial role in cell proliferation and survival [154]. Glutamine is a non-essential amino acid that is critical for the growth of cancer cells. Glutamine is converted to glutamate by glutaminase, and glutamate is further metabolized to α-KG, which feeds into the TCA cycle to generate energy and biosynthetic precursors [155]. In vitro analyses of different cancer cell lines have indicated the glutamine dependency of many SPCs [156]. In SPCs, such as ovarian, colorectal, and gastric cancers, metabolic alterations contribute to the development of chemoresistance and cancer progression. The inhibition of glutamine metabolism has been shown to have antitumor effects in preclinical models of these cancers [157,158]. In ovarian cancer, which frequently metastasizes to the peritoneum, glutamine dependency has been shown to promote chemoresistance [159]. Increased expression of glutaminase and the glutamine transporter SLC1A5/ASCT2 has been observed in cisplatin-resistant ovarian cancer cells, and the inhibition of ASCT2 and glutaminase has been shown to sensitize these cells to cisplatin [160]. Inhibiting ASCT2 has also been shown to reduce the growth and metastasis of pancreatic cancer cells [108]. Several preclinical studies have indicated that a combination therapy with glutaminase inhibitors could provide a survival advantage to cancer patients [156,161]. For instance, it has been observed that treatment with the glutaminase inhibitor 968 along with anti-PD-L1 reduced the intraperitoneal dissemination of ovarian cancers [109]. Glutaminase-1 inhibitor, used in combination with doxorubicin, has been shown to override the doxorubicin resistance of pancreatic adenocarcinoma ascites metastasis cells [110]. Furthermore, inhibiting the glutamine pathway sensitized the cancer cells to gemcitabine therapy in pancreatic cancers [162]. In addition, cisplatin resistance in ovarian cancers has been reported to be overcome by the inhibition of glutamate dehydrogenase, which catalyzes the glutamate to α-KG conversion to fuel the TCA cycle [163,164]. 

In colorectal cancer, which can also metastasize to the peritoneum, a dynamic metabolic crosstalk involving cancer cells and peritoneal adipocytes that promotes chemoresistance has been observed. Cancer cells override adipocytes for the uptake of glutamine from the PTME, and peritoneal adipocytes enhance endogenous glutamine synthesis to overcome the glutamine shortage. This process is mediated by the epigenetic de-repression of the gene GS, which encodes glutamine synthetase [72]. Increased glutamine levels in the peritoneal adipocytes lead to the release of glutamine to the PTME, replenishing the glutamine pool. Glutamine from the glutamine pools is taken up by the cancer cells for the subsequent utilization in bioenergetic and biosynthetic pathways within the cancer cells [165,166]. 

Another amino acid metabolic pathway that has been validated as a target for cancer therapy is the methionine metabolic pathway [167]. Methionine is an essential amino acid that is converted to S-adenosylmethionine (SAM), which serves as a methyl donor in numerous cellular processes, including DNA methylation and protein methylation. Methionine metabolism is critical for cancer cells, as it is required for the synthesis of polyamines, which are necessary for cell proliferation [168]. The inhibition of methionine metabolism has been shown to have antitumor effects in multiple preclinical models of different tumors, including the ones that can metastasize into the peritoneum [111,169]. In addition, it has been shown that the combination of a methionine-restricted diet with chemotherapy had a greater antitumor effect when compared with chemotherapy alone [170]. Arginine metabolism is also emerging as a potential target for cancer therapy [171]. Arginine is an essential amino acid that plays a crucial role in immune function, and its depletion has been shown to impair the growth and survival of cancer cells. Induced depletion of arginine has been shown to inhibit the proliferation and invasive migration of many cancers that reside in the peritoneum such as ovarian, pancreatic, and liver cancers [172]. The metabolic enzyme, arginase, which catalyzes the conversion of arginine to ornithine and urea, is also being targeted for therapeutic strategy [112,171]. Dysregulated expression of arginase resulting in its upregulation is observed in many cancers [112]. Inhibition of arginase has been shown to reduce tumor burden in preclinical models of colorectal, breast, lung, and ovarian cancers as well as hepatocellular carcinoma [112]. Arginine metabolism also involves the synthesis of polycationic polyamines that are considered oncometabolites in colon and colorectal cancers [113,173,174]. Increased levels of polyamines have been correlated with the proliferation and survival of colorectal cancer cells [113,173,174]. However, their role in the context of PPCs and SPCs remains to be established.

Another potential target in amino acid metabolism is the serine-glycine one-carbon metabolism pathway, which plays a critical role in nucleotide synthesis and DNA methylation [175,176]. Inhibition of this pathway has been shown to have anti-tumor effects in several types of cancers, including those of liver, colorectal, breast, and ovarian cancers [177,178,179,180]. Sarcosine or N-methyl glycine is a potential oncometabolite synthesized from glycine. It has been observed that the levels of sarcosine are elevated during metastasis of cancers of extra-peritoneal origin such as gastric and colorectal cancers [114,181]. Though sarcosine upregulation in peritoneal cancers hasn’t been reported yet, its potential role in tumor progression and as a biomarker makes it a potential oncometabolite candidate for peritoneal cancers that could be explored further. 

LC-MS analysis of peritoneal lavage fluid from the peritoneal metastases of gastric cancer patients has identified several upregulated amino acid metabolites including 3-methyl alanine, glutamyl alanine, and α-amino butyric acid [49]. Among these metabolites, α-amino butyric acid has been reported to be elevated in diverse cancers, such as ovarian and colorectal cancers, metastasizing to the peritoneum. It has also been associated with oxidative stress-induced glutathione metabolism in peritoneal cancers. Targeting α-amino butyric acid metabolism has shown promise in reducing tumor growth and metastasis in colorectal cancer models [63]. Inhibiting the synthesis of α-aminobutyric acid metabolism, using 4-aminobutyrate aminotransferase (ABAT), has been shown to reduce the proliferation and migration of colorectal cancer cells [115].

Resistance to chemotherapy is a major challenge in the treatment of peritoneal cancers. One potential mechanism of chemotherapy resistance is the upregulation of amino acid metabolic pathways, which can lead to increased tumor growth and survival. When the expression of amino acid metabolic enzymes was evaluated in samples of peritoneal cancer tissue from patients who had received chemotherapy, it was observed that the expression of glutaminase and methionine adenosyltransferase was significantly higher in patients who had developed resistance to chemotherapy compared to those who had responded to the treatment [117]. Overall, these studies provide evidence for the potential efficacy of targeting amino acid metabolism in peritoneal cancer therapy. While further research is needed to develop effective therapies and identify optimal treatment strategies, targeting amino acid metabolism remains a promising approach for the treatment of peritoneal cancer.

Clinical studies have also identified oncometabolites generated from amino acid metabolism as potential biomarkers for predicting the treatment response and prognosis in peritoneal cancers. For example, elevated levels of 2-HG have been associated with poor prognosis in patients with PCs originating from colon and colorectal cancers [130,182]. Similarly, elevated levels of homocysteine have been shown to be a potential biomarker for digestive tract cancer risk [183]. 

### 3.4. Targeting Nucleotide Metabolism

An obvious and clinically utilized example for targeting nucleotide metabolism for chemotherapy is the use of nucleoside analogs that mimic the structure of nucleotides and can be incorporated into DNA or RNA, disrupting their normal function. Several key nodes of nucleotide metabolism have been investigated for their potential as therapeutic targets for peritoneal cancers [92]. The inhibition of thymidylate synthase, involved in the synthesis of thymidine, has been shown to be effective in the treatment of several types of cancers, including colorectal cancer [116,184]. It has been observed that treatment with pemetrexed, an inhibitory analog of folate, elicits significant therapeutic efficacy in recurrent peritoneally metastasized ovarian cancers [118]. Additionally, the combination of pemetrexed with cisplatin resulted in a greater antitumor effect when compared to either of the drugs alone in peritoneal mesothelioma [117,185,186]. Another enzyme involved in nucleotide metabolism that has been proposed as a target for peritoneal cancer therapy is dihydrofolate reductase (DHFR). DHFR is involved in the conversion of dihydrofolate into tetrahydrofolate, a co-factor required for nucleotide synthesis. Inhibition of DHFR has been shown to be effective in the treatment of several types of cancers [119]. It is significant to note that Raltitrexed and Pemetrexed, two DHFR inhibitors, have been clinically approved for colorectal cancer and pleural mesothelioma, respectively [119]. Their clinical efficacy in peritoneal mesothelioma and other peritoneal cancers remains to be evaluated. The inhibition of inosine monophosphate dehydrogenase (IMPDH) is another potential strategy for targeting nucleotide metabolism in cancer therapy [120]. IMPDH is involved in the de novo synthesis of guanine nucleotides, which are necessary for DNA and RNA synthesis. While experimental studies have shown that the inhibition of IMPDH could inhibit the proliferation of cells from multiple cancers, its therapeutic efficacy in SPCs remains to be established [119]. 

Another potential target in nucleotide metabolism is ribonucleotide reductase (RNR), which catalyzes the conversion of ribonucleotides into deoxyribonucleotides, the building blocks of DNA [121]. It has been observed that the expression of RNR was significantly higher in patients who had developed resistance to chemotherapy when compared with those who had responded to the treatment [187]. Several RNR inhibitors have been clinically approved as single-agent therapy for different cancers including pancreatic cancer [121]. Gemcitabine, an RNR inhibitor, has already been used in a single-agent therapeutic regimen for platinum-resistant ovarian cancers, fallopian tube cancer, and primary peritoneal adenocarcinoma [122]. In addition, gemcitabine has been shown to reverse cisplatin resistance in ovarian and peritoneal carcinomas [188]. Treatment with nucleoside analogs such as gemcitabine and inhibitors of nucleotide metabolism remains the current standard of care for PPCs and SPCs [189]. A phase II clinical trial with a combination therapy involving gemcitabine and the topoisomerase inhibitor, irinotecan, led to a disease control rate of 75% with increased progression-free survival of platinum-refractory ovarian or peritoneal cancer patients [123]. While further research is needed to develop effective therapies and identify optimal treatment strategies, targeting nucleotide metabolism remains a promising approach for the treatment of peritoneal cancers.

## 4. Conclusions and Perspectives 

The identification and characterization of oncometabolites in peritoneal cancers represent a promising area of research for developing precision cancer medicine strategies. With cancer being a heterogenous disease, identifying a specific target for each individual patient can be challenging. The vast metabolic diversity of cancer cells poses a challenge in identifying specific targets for precision cancer medicine strategies. Nonetheless, with the development of advanced analytical techniques and bioinformatics tools, it is becoming increasingly feasible to analyze large-scale metabolomic data and identify key metabolites and metabolic pathways that drive cancer progression. Such information can be used to design targeted therapies that disrupt the specific metabolic vulnerabilities of cancer cells while minimizing toxicity to normal tissues. Moreover, the integration of oncometabolite profiling into clinical practice may help to guide treatment decisions and improve patient outcomes. For example, oncometabolite profiling could be used to identify patients who are most likely to benefit from specific targeted therapies or combination treatments. In addition, oncometabolite profiling may help to identify new drug targets and pathways for developing novel therapeutic approaches for peritoneal cancers. 

With the advancement of metabolomics technologies and bioinformatics tools, it is becoming increasingly feasible to profile oncometabolites in patient samples and develop personalized treatment plans based on the specific metabolic profile of each patient’s tumor. Despite its potential benefits in precision cancer medicine, oncometabolite profiling still faces several challenges that must be overcome before it can be widely implemented in clinical practice. The lack of standardized protocols for sample collection and processing is one of the main hurdles, as it can compromise the accuracy and reproducibility of results. Additionally, the identification of rare or novel metabolites through oncometabolite profiling poses a challenge for the development of targeted therapies, as many of these metabolites have yet to be targeted by drugs. While 2-HG has already been successfully targeted, new therapies will need to be developed for other oncometabolites. Finally, larger clinical trials that carefully control for confounding factors will be necessary to validate the utility of oncometabolites as biomarkers for therapeutic response and prognosis. Such trials must involve a significant number of patients to generate meaningful results [12].

In conclusion, the identification and characterization of oncometabolites in peritoneal cancers provide a promising avenue for developing precision cancer medicine strategies. Preclinical and clinical studies have demonstrated the potential of targeting specific oncometabolites in peritoneal cancers, and future research in this area may lead to the development of novel therapeutic approaches that can improve patient outcomes. SPCs have provided important insights into the molecular mechanisms and metabolic pathways involved in peritoneal cancers and can inform the development of new therapeutic approaches for these cancers. By integrating oncometabolite profiling into clinical practice, it may be possible to develop more effective and personalized therapeutic strategies for patients with peritoneal cancers. 

Finally, the use of oncometabolite profiling in combination with other biomarkers, such as genetic mutations and immune markers, may further improve the accuracy of patient stratification and personalized treatment planning. For example, the combination of oncometabolite profiling and immune marker profiling may help to identify patients who are most likely to respond to immunotherapy, which has shown promise in the treatment of several types of cancers. Ongoing and future research in this area is focusing on developing new methods for profiling the metabolic activity of peritoneal tumors, identifying additional oncometabolites that are specific to these tumors, and validating the utility of oncometabolites as biomarkers for treatment response and prognosis. However, there is still much more to be learned about the metabolic profile of peritoneal cancers and how oncometabolites contribute to tumor growth and metastasis. Future research in this area should focus on identifying additional oncometabolites that are specific to peritoneal cancers and developing new methods for profiling the metabolic activity of these tumors. The integration of oncometabolite profiling into clinical practice may help to guide treatment decisions and improve patient outcomes.

## Figures and Tables

**Table 1:** Targeting of oncometabolites and metabolic pathways in PPCs and SPCs.

S. No.	Target Molecule	Targeted Pathway	Drug/Inhibitor	Cancer Type	Reference
**Targeting Glucose Metabolism**					
1.	Glu	Glycolysis	2-deoxyglucose	PPC; OvCa	[95]
2.	HK2	Glycolysis	3-bromopyruvate	PPC	[96]
3.	LDH	Glycolysis	LDH Inhibitor	PPC	[97]
4.	PGK1	Glycolysis	5-FU + shRNA-PGK1	PPC; GC	[98]
5.	2HG	TCA Cycle	IDH1 Inhibitor	PPC; OvCa	[99]
6.	G6PD	PPP	G6PD Inhibitor	BrCa; OvCA; PaCa	[100]
7.	6PGD	PPP	6PGD Inhibitor	OvCa; HCC	[101]
**Targeting Lipid Metabolism**					
8.	CD36	FA uptake	CD36 Inhibition	OvCa	[102]
9.	FABP4	FA uptake	FABP4 Inhibitor	OvCa	[102]
10.	ACC	FA synthesis	ACC Inhibitor	BrCa; CCC	[103]
11.	FASN	FA synthesis	Orlistat, Cerulenin	PrCa; CC	[104,105]
12.	CS	Sphingolipid synthesis	CS Inhibitor	OvCa; BrCa; CC	[106]
13.	SPK	Sphingolipid synthesis	SPK Inhibitor	BrCa; PrCa; CC	[107]
**Targeting Amino Acid Metabolism**					
14.	ASCT2	Glutamine uptake	ASCT2 Inhibitor	PaCa; OvCa	[108]
15.	Glutaminase	Glutaminolysis	968 + Anti-PD-L1, Glutaminase Inhibitor + Doxorubicin	OvCa, PaCa	[109,110]
17.	Met	Met-metabolism	Met-restriction	BrCa; CC; PrCa	[111]
18.	Arginase	Arg-metabolism	Arginase Inhibitor	CRC; BrCa; LC; OvCa; HCC	[112]
19.	Polycationic polyamines	Arg-metabolism	Polyamine Inhibitor	CC; CRC	[113]
20.	Sarcosine	Ser-Gly metabolism	Sarcosine Inhibitor	PrCa; GC; CRC	[114]
21.	α-Amino butyric acid	Met/Ser/Thr-metabolism	ABAT	CRC	[115]
**Targeting Nucleotide Metabolism**					
22.	TYMS	Purine & Pyrimidine	ZD9331, AG337	CRC; CC	[116]
23.	DHFR	Purine & Pyrimidine	Pemetrexed, Raltitrexed	PPC; Ovary; CRC	[117,118,119]
25.	IMPDH	Purine	Tiazofurin	PaCa; OvCa; CC; BrCa	[120]
26.	RNR	Purine & Pyrimidine	Gemcitabine	OvCa; FTC; PPC	[121,122,123]

Abbreviations used are 2HG, 2-hydroxyglutarate; 5-FU, 5-fluorouracil; 6PGD, 6-phosphogluconate dehydrogenase; ABAT, 4-aminobutyrate aminotransferase; ACC, Acetyl CoA carboxylase; ASCT, 2-Amino acid transporter Alanine Serine Cysteine transporter-2; BrCa, Breast Cancer; CC, Colon Cancer; CCC, Cholangiocarcinoma; CD36, Fatty acid translocase CD36; CRC, Colorectal Cancer; CS, Ceramide synthase; DHFR, Dihydrofolate reductase; FA, Fatty acids; FABP4, Fatty acid binding protein 4; FASN, Fatty acid synthase; FTC, Fallopian Tube Cancer; GC, Gastric Cancer; G6PD, glyceraldehyde-6-phosphate; HCC, Hepatocellular carcinoma; HK2, Hexokinase 2; IDH1, Isocitrate dehydrogenase 1; IMPDH, Inosine monophosphate dehydrogenase; LDH, Lactate dehydrogenase; LC, Lung Cancer; OvCa, Ovarian Cancer; PaCa, Pancreatic Cancer; PD-L1, Programmed death ligand-1; PGK1, Phosphoglycerate kinase 1; PPC, Primary Peritoneal Cancer; PPP, Pentose phosphate pathway; PrCa, Prostate Cancer; RNR, Ribonucleotide reductase; SPK, Sphingosine kinase; TCA, Tricarboxylic acid cycle.
